# Machine Learning for the Detection and Diagnosis of Anomalies in Applications Driven by Electric Motors

**DOI:** 10.3390/s23249725

**Published:** 2023-12-09

**Authors:** Fábio Ferraz Júnior, Roseli Aparecida Francelin Romero, Sheng-Jen Hsieh

**Affiliations:** 1 Mechatronic Engineering, INSPER—Institute of Education and Research, São Paulo 04546-042, SP, Brazil; 2 Institute of Mathematical and Computer Sciences, University of Sao Paulo, Sao Carlos 13566-590, SP, Brazil; rafrance@icmc.usp.br; 3 Engineering Technology & Industrial Distribution (Joint Appt with Mechanical Engineering), Texas A&M University, College Station, TX 77840, USA; hsieh@tamu.edu

**Keywords:** machine learning, artificial intelligence, electric motor, rotating machinery, predictive maintenance, condition monitoring

## Abstract

Manufacturing systems are becoming increasingly flexible, necessitating the adoption of new technologies that allow adaptations to a turbulent and complex modern market. Consequently, modern concepts of production systems require horizontal and vertical integration, extending across value networks and within a factory or production shop. The integration of these environments enables the acquisition of a substantial amount of data containing information pertaining to production, processes, and equipment located on the shop floor. When these data and information are processed and analyzed, they have the potential to reveal valuable insights and knowledge about the manufacturing systems, offering interpretive outcomes for strategic decision making. One of the opportunities presented in this context includes the implementation of predictive maintenance (PdM). However, industrial adoption of PdM is still relatively low. In this paper, the aim is to propose a methodology for selecting the main attributes (variables) to be considered in the instrumentation setup of rotating machines driven by electric motors to decrease the associated costs and the time spent defining them. For this, the most well-known data science and machine learning algorithms are investigated to choose the one most adequate for this task. For the experiments, different testing scenarios were proposed to detect the different possible types of anomalies, such as uncoupled, overloaded, unbalanced, misaligned, and normal. The results obtained show how these algorithms can be effective in classifying the different types of anomalies and that the two models that presented the best accuracy values were k-nearest neighbor and multi-layer perceptron.

## 1. Introduction

Manufacturing systems are becoming increasingly flexible, necessitating the adoption of new technologies that allow adaptations to a turbulent and complex modern market (Salazar et al., 2019 [1]; Hozdić, 2015 [2]). Consequently, modern concepts of production systems require horizontal and vertical integration, extending across value networks and within a factory or production shop (Salazar et al., 2019 [1]; Hozdić, 2015 [2]). The necessity to adapt and employ new technologies to promote the integration of production systems has propelled the industry into a new era, referred to as Industry 4.0. (Hozdić, 2015 [2]; Zonta et al., 2020 [3]). The integration of these environments enables the acquisition of a substantial amount of data containing information pertaining to production, processes, and equipment located on the shop floor. When these data and information are processed and analyzed, they have the potential to reveal valuable insights and knowledge about manufacturing systems, offering interpretive outcomes for strategic decision making (Carvalho et al., 2019 [4]). One of the opportunities presented in this context includes the implementation of predictive maintenance (PdM), mainly within the context of Industry 4.0 and its enabling technologies (Zenisek et al., 2019 [5]; Sarmiento et al., 2020 [6]). PdM enables the early detection of failures using predictive tools based on historical data that employ machine learning techniques, thus enabling maintenance execution only when necessary (Carvalho et al., 2019 [4]; Zenisek et al., 2019 [5]; Cakir et al., 2021 [7]). According to Cavalieri and Salafia (2020) [8], one-third of all maintenance costs are wasted due to unnecessary maintenance or incorrect execution. Industry 4.0, characterized by its key technologies such as the Internet of Things (IoT); big data; and artificial intelligence (AI), specifically data science and machine learning (ML) algorithms, has facilitated the transformation of the traditional paradigm. An illustration of this shift involves the adoption of predictive maintenance (Caldana et al., 2021 [9]).

In this paper, the following objectives are defined:

- To apply data science and ML algorithms targeting the PdM of specific industrial equipment to assess their effectiveness in detecting anomalies.
- To verify whether data science and ML algorithms can help define the instrumentation configuration in a PdM application.

Lee et al. (2019) [10] claimed that the emergence of Industry 4.0 is directing more focus toward PdM strategies aimed at reducing downtime costs and enhancing the availability of manufacturing equipment. PdM uses time-based information and knowledge to report a possible failure, thereby preventing downtime. This has been made possible by incorporating technologies into the industrial environment, such as the Industrial Internet of Things (IIoT), machine learning, and big data (Zonta et al., 2020 [3]). The recent advancements in industrial artificial intelligence have shown its potential to help manufacturers navigate the challenges of the digital transformation of cyber-physical systems through its predictive analysis based on data and ability to assist decision making in highly complex processes (Peres et al., 2020 [11]). However, according to Peres et al. (2020) [11], the industrial implementation of such solutions remains relatively low beyond the experimental pilot phase, given that real-world environments represent unique and difficult challenges for which organizations are not yet adequately prepared. Corroborating this perception, Zenisek et al. (2019) [5] stated that real-world implementations of PdM remain infrequent due to the scarcity of high-quality monitoring data and little experience regarding the applicability of analysis methods.

In this context, the main contribution of this paper is the methodology used to select the variables for consideration in the instrumentation configuration of machines based on data science and machine learning (ML) algorithms. We will show that these algorithms are effective in determining the number of sensors to be adopted to detect anomalies in machines, implying a decrease in the associated costs and time involved in defining the instrumentation setup. Furthermore, we build a database containing registers of the different scenarios of applications driven by electric motors: normal operating conditions and four anomaly scenarios (uncoupled, overloaded, unbalanced, and misaligned).

This article is organized as follows. Section 2 presents some topics relevant to the subject discussed here, such as industrial maintenance, maintenance strategies, machine learning for PdM, and applications in rotating machines. Section 3 describes the proposed methodology, resources, and technologies, as well as the test scenarios proposed by us for PdM applications. Section 4 presents the experiments and results of implementing data science and ML algorithms for PdM. Finally, Section 5 presents the conclusions, limitations, and suggestions for future work.

## 2. Related Works

This section presents a review of issues relevant to the development of the present article: industrial maintenance, machine learning for predictive maintenance, and anomaly detection.

### 2.1. Industrial Maintenance

The standard BS EN 13306:2017 [12] defines maintenance as the “combination of all technical, administrative and managerial actions during the life cycle of an item intended to retain it in, or restore it to, a state in which it can perform the required function”. The need for maintenance is based on actual or impending failures (FEMP, 2010 [13]).

**Figure 1:**
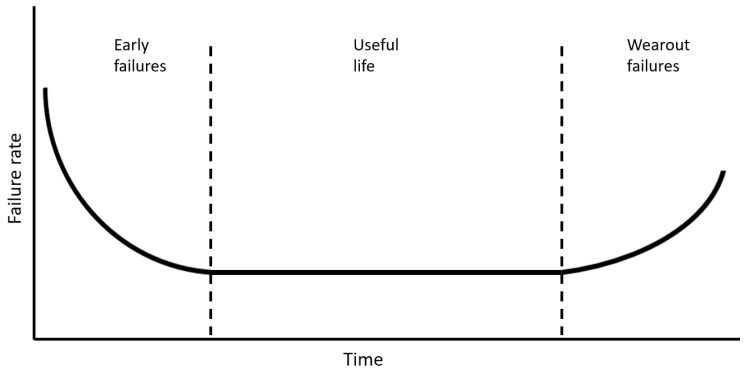
Failure rates over time.

The literature (e.g., FEMP, 2010 [13]; NASA, 2008 [14]; Smith, 2022 [15]; Souza, 2021 [16]) indicates that a graphical representation of the failure rate of a population of components versus time would probably be in the form of a “bathtub”, as shown in Figure 1. In this representation, the Y-axis indicates the rate of failure, whereas the X-axis corresponds to time. Based on its shape, the curve can be divided into three distinct zones: early failures, useful life, and wear-out failures (FEMP, 2010 [13]; NASA, 2008 [14]; Smith, 2022 [15]; Souza, 2021 [16]).

According to FEMP (2010) [13], NASA (2008) [14], Smith (2022) [15], and Souza (2021) [16], the early period, i.e., the infant mortality region of the bathtub curve, is characterized by a high failure rate followed by a period of decreasing failure. Many of the faults associated with this region are related to inappropriate projects and poor manufacturing and assembly, including human error during installation and operation (FEMP, 2010 [13]; Souza, 2021 [16]). The period of infant mortality is followed by a nearly constant period known as useful life (FEMP, 2010 [13]; NASA, 2008 [14]; Smith, 2022 [15]; Souza, 2021 [16]). There are many theories about why components fail in this period, but most authors have recognized that poor operation and maintenance often play a significant role (FEMP, 2010 [13]). It is also generally accepted that practices of exceptional maintenance, including preventive and predictive elements, can extend this period (FEMP, 2010 [13]). Finally, the wear-out period is characterized by a rapid increase in the rate of failure over time (FEMP, 2010 [13]; NASA, 2008 [14]; Smith, 2022 [15]; Souza, 2021 [16]). In most cases, this period encompasses the normal distribution of failures over the lifetime of a project (FEMP, 2010 [13]).

The lifetime of most equipment necessitates periodic maintenance. Moreover, when we fail to perform the maintenance activities intended by the equipment designer, we reduce the useful life of the equipment (FEMP, 2010 [13]; NASA, 2008 [14]; SMITH, 2022 [15]).

#### Maintenance Strategies

Various classifications of maintenance strategies can be found in the literature. The following are the maintenance methods most commonly cited:

- Reactive Maintenance: This is also known as corrective maintenance or run-to-failure maintenance mode. According to the standard BS EN 13306:2017 [12], reactive maintenance is performed after fault recognition and is aimed at restoring an item to a state where it can perform a necessary function. Exclusively relying on reactive maintenance often results in a substantial number of unplanned maintenance tasks, elevated inventories of spare parts, and inefficient allocation of maintenance resources (NASA, 2008 [14]).
- Preventive Maintenance: This is performed according to established time intervals or the number of units in use but without prior investigation of the condition of the system (BS EN 13306:2017 [12]). While preventive maintenance may not be the ideal maintenance approach, it offers several advantages compared to a purely reactive program. For instance, it helps ensure the system’s designed lifespan and reduces the frequency and severity of unplanned machine failures (FEMP, 2010 [13]; NASA, 2008 [14]).
- Predictive Maintenance (PdM): This is also known as condition-based maintenance. PdM is performed according to forecasts extrapolated from repeated analyses or known characteristics and evaluations of significant parameters related to the degradation of an item (BS EN 13306:2017 [12]). PdM is conducted according to the actual condition of the system, rather than a predefined schedule like preventive maintenance (FEMP, 2010 [13]). According to Theissler et al. (2021) [17], PdM aims to predict the ideal time for maintenance actions, taking into account information about the state of health of the system and/or data maintenance history. Furthermore, according to the same author, it aims to prevent early and expensive repairs by ensuring timely maintenance before any failures occur.

According to several authors, such as Zhang and Yang (2019) [18], Chen et al. (2020a) [19], Zonta et al. (2020) [3], and Cakir et al. (2021) [7]), PdM has become the most effective solution in the industry due to reduced maintenance costs, reduced equipment downtime, and reduced probability of accidents, as it guarantees safe operations.

With this type of maintenance, it is essential to accurately predict the next failure. There exist various approaches for PdM modeling, including statistical approaches and machine learning. Recently, machine learning models have been widely used in PdM, achieving satisfactory performance. According to Nacchia et al. (2021) [20], PdM has shown great potential when guided by an ML algorithm. Several studies have corroborated this statement, such as those by Zhang and Yang (2019) [18]; Chen et al. (2020a) [19]; Nentwich, Junker, and Reinhart (2020) [21]; Martins, Rodriguez, and Henriques (2020) [22]; Welte, Estler, and Lucke (2020) [23]; and Theissler et al. (2021) [17]. Thus, the next section presents the relevant fundamentals of ML for PdM applications.

### 2.2. Machine Learning for Predictive Maintenance

Fundamentally, machine learning involves building models and equations to help make sense of data. “Learning” is achieved by adjusting the parameters of these models from training data. These adjusted models can be used to predict and understand aspects of newly observed data (Vanderplas, 2016 [24]).

Recently, machine learning has been widely used in different applications in industry, such as equipment maintenance operations (Chen et al., 2020a [19]; Theissler et al., 2021 [17]). According to Welte, Estler, and Lucke (2020) [23], many companies see AI, in particular machine learning, as an important strategic component that can be used to obtain competitive advantages. The same authors concluded that machine learning algorithms have seen significant advancements in performance and applicability in industrial maintenance in recent years. Machine learning applications allow for predictive maintenance and, therefore, can increase efficiency. PdM methods are mainly divided into the following three categories (Zhang and Yang, 2019 [18]):

- Model-based prognosis;
- Knowledge-based prognosis;
- Data-based prognosis.

Notably, data-based prognosis has attracted a great deal of attention. In recent years, the data-based approach has gained popularity due to its high practicality, as it incorporates artificial intelligence (Lang et al., 2021 [25]).

**Figure 2:**
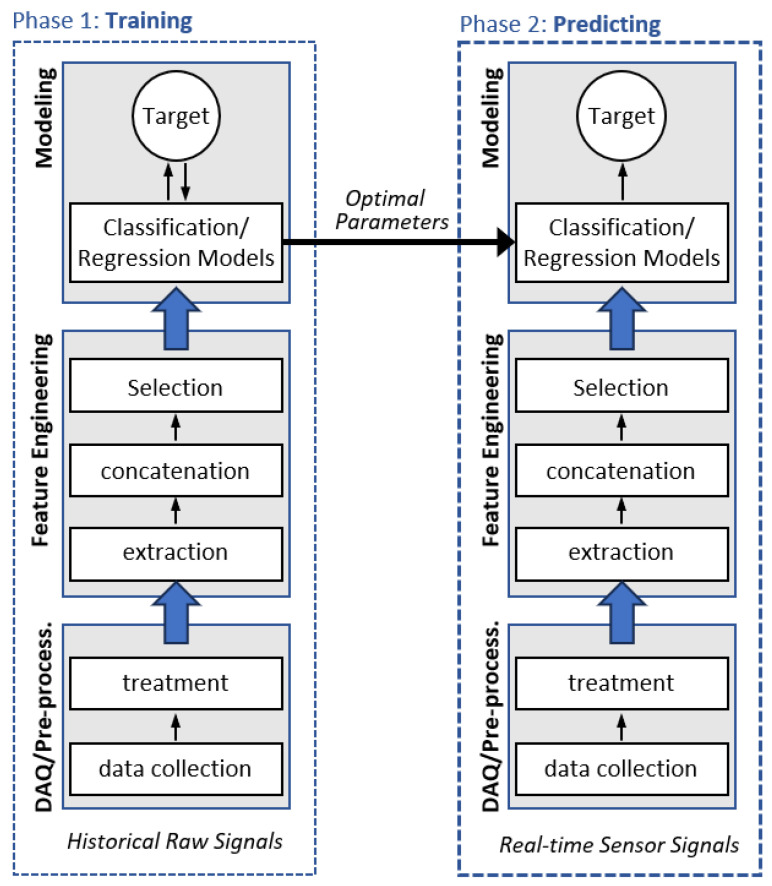
PdM method based on data (modified from [18]).

According to Zhang and Yang (2019) [18], data-driven predictive maintenance consists of two phases, as shown in Figure 2. First, a learning process (i.e., model training) is required based on historical raw sensor signals, and second, the trained model is applied to predict targets and make decisions.

In general, each phase consists of the following three sub-processes (Zhang and Yang, 2019 [18]):

- Data acquisition and pre-processing: data collection and treatment, either monosensory or multisensory;
- Feature engineering: extraction, concatenation, and selection of attributes/characteristics representative of the system’s condition;
- Model training and prediction: the model is generated and optimized for use in forecasts.

After completing these phases and sub-processes, the model can be used to carry out predictions based on real-time data flow (ZHANG and YANG, 2019 [18]).

**Figure 3:**
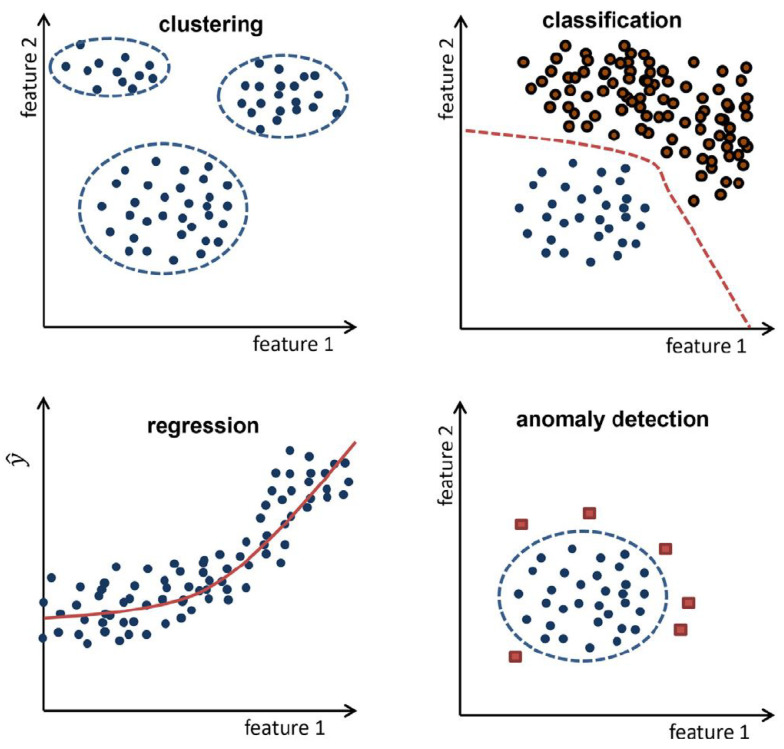
Relevant tasks for PdM [17].

According to Theissler et al. (2021) [17], the most relevant machine learning tasks for PdM applications are (i) clustering, (ii) classification, (iii) regression, and (iv) anomaly detection (illustrated in Figure 3).

Regarding anomaly detection, the objective of PdM is intricately linked with modeling a system’s normal behavior and identifying deviations, commonly referred to as anomalies, that could indicate current or developing failures (see lower right corner of Figure 3) (Theissler et al., 2021 [17]). Furthermore, according to Theissler et al. (2021), “Anomaly detection is a common approach for fault detection. Within the taxonomy of error, fault, and failure, an anomaly can be considered as a potential error, where an error is caused by a fault and may in turn cause a failure”.

Fault detection and diagnosis (FDD) is a conditional monitoring technique used to detect faults and distinguish different types of faults to make decisions in advance, avoiding dangerous occurrences (Lang et al., 2021 [25]). Thus, anomaly detection can point to a failure and, therefore, can be used for condition-based PdM (Theissler et al., 2021 [17]).

#### Applications in Rotating Machines

Rotating machines are among the most important pieces of equipment in modern industrial applications (Liu et al., 2018 [26]). In these machines, electric motors represent one of the most critical components for converting electrical energy into mechanical energy. For instance, the induction motor has been widely employed in various industrial processes due to its cost-effectiveness, reliability, and robustness (Alshorman et al., 2020 [27]). Initially, manufacturers and users of electrical machines primarily depended on basic protection like overcurrent and overvoltage protection, as well as ground-fault protection, to guarantee the safe and dependable functioning of these systems (Nandi et al., 2005 [28]). Nevertheless, with the growing complexity of the tasks executed by these machines, there arose a demand for enhancements in the realm of fault diagnosis (Nandi et al., 2005 [28]).

Fault detection and diagnosis can be employed to assess the current operational state of an electric motor, enabling the early detection of issues and predictive analysis (Lang et al., 2021 [25]; Liu et al., 2018 [26]; Alshorman et al., 2020 [27]; Nandi et al., 2005 [28]). According to Liu et al. (2018) [26] and Alshorman et al. (2020) [27], addressing issues with rotating machinery emerges as the pivotal element in system design and maintenance. This encompasses the processes of detecting, isolating, and identifying faults, thereby providing valuable insights into the equipment’s operational status. These tasks can be categorized into three fundamental fault diagnosis objectives (Liu et al., 2018 [26]):

- To determine whether the equipment is normal;
- To find the incipient failure and its cause;
- To forecast the trend of fault development.

**Figure 4:**
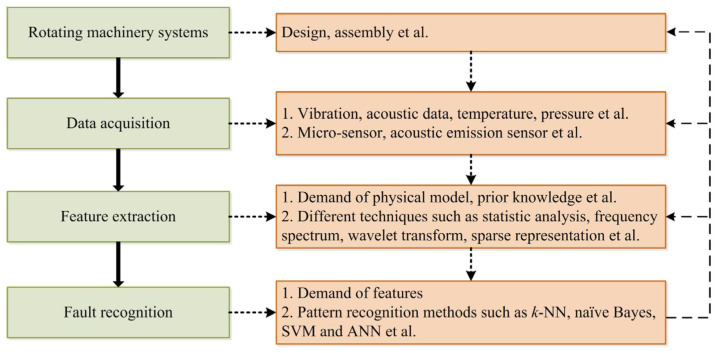
Fault diagnosis framework for predictive maintenance [26].

Therefore, fault diagnosis can be essentially thought of as a pattern recognition problem regarding the state of rotating machines (Liu et al., 2018 [26]). Moreover, artificial intelligence (AI), serving as a potent tool for pattern recognition, has garnered significant interest among many researchers and holds potential for use in the field of fault detection in rotating machinery. Specifically, classifiers and statistical learning methods have been widely used in the fault diagnosis of rotating machinery, including the k-nearest neighbor (k-NN) algorithm, naive Bayesian classifier (NB), support vector machine (SVM), and multi-layer perceptron (MLP) (Lang et al., 2021 [25]; Liu et al., 2018 [26]; Alshorman et al., 2020 [27]; Dias, 2019 [29]). Figure 4 shows a standard fault diagnosis framework for the operation and maintenance of engineering systems.

## 3. Methodology

**Figure 5:**
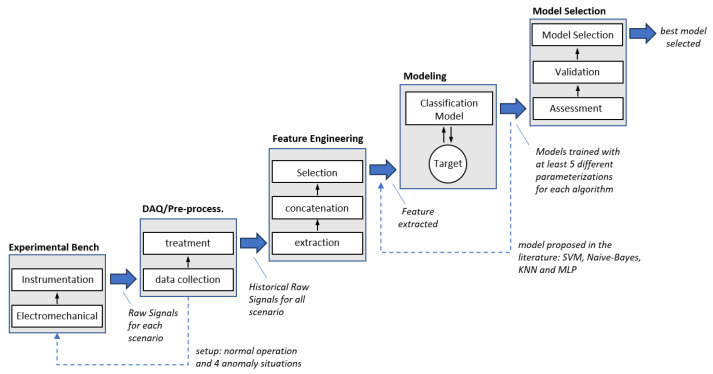
Experimental procedure.

For the development of the present article, a methodological procedure was proposed, as illustrated in Figure 5, where:

- Experimental Bench: Prepare the experimental bench to generate datasets encompassing 5 distinct scenarios in applications powered by electric motors: normal operating conditions and four anomaly scenarios. These various scenarios correspond to the dataset classes representing anomalies that can occur.
- DAQ/Pre-Processing: Collect data from various sources, perform data cleansing, and convert the data into a usable format.
- Feature Engineering: Based on the literature, generate attributes from signals collected in the previous step, concatenate them, and select relevant attributes.
- Modeling: Select classifier models from the literature and optimize them by applying a minimum of 5 different parameterizations for each algorithm.
- Model Selection: Evaluate classifier performance and select the best model.

The stages of this methodological procedure provide the framework for executing the experiments and analyzing the results presented in subsequent sections.

### 3.1. Resources and Technologies

Resources and technologies were employed for setting up the experimental bench and implementing the machine learning techniques. The bench was developed to simulate scenarios in applications of industrial units driven by electric motors for rotating machinery. It can be subdivided into an electromechanical device and instrumentation system.

#### 3.1.1. Electromechanical Device

**Figure 6:**
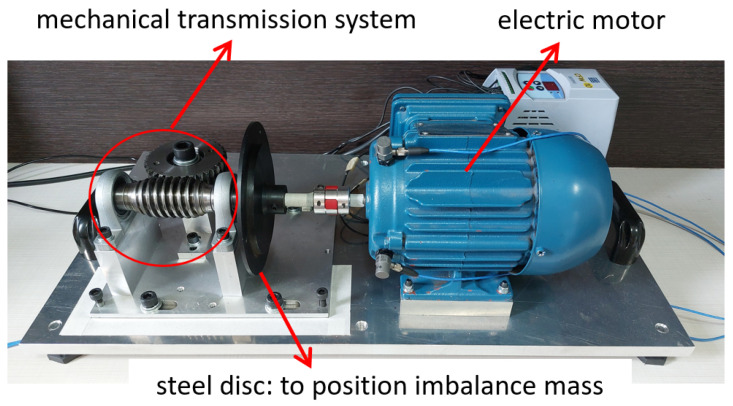
Electromechanical device of the experimental bench.

The electromechanical device used was based on the test bench presented by Dias (2019) [29], with modifications mainly to the type of engine used and the method of applying overload in the electromechanical system. It consists of a worm-gear transmission system with a crown gear coupled to the shaft of an electric cage motor. Between the mechanical transmission system and the electric motor, a steel disc with a diameter of 130 mm was installed. This steel disc was adopted to position an imbalance mass 60 mm from the center of the shaft. Figure 6 shows the electromechanical device.

The electric motor used was a 0.25 kW, 220 VAC, three-phase cage induction motor (WEG manufacturer, Brazil, W22 model, 1710 Hz nominal speed). The motor drive was a frequency inverter (WEG manufacturer, Brazil, CFW10 EasyDrive model, CFW100026S2024PSZ).

#### 3.1.2. Instrumentation

**Figure 7:**
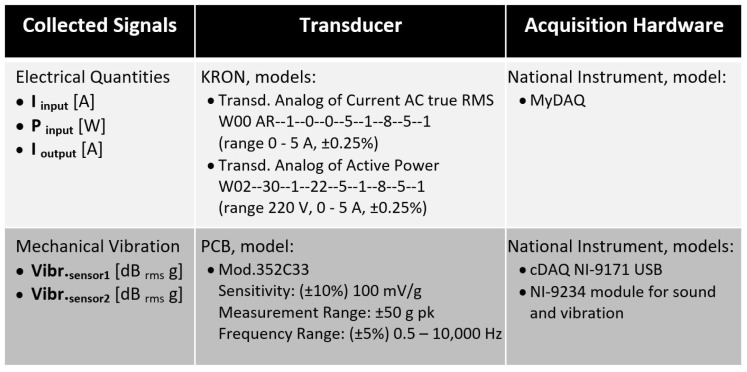
Data acquisition hardware.

The instrumentation consisted of transducers and data acquisition hardware, as illustrated in Figure 7. These components were employed for collecting data on electrical magnitudes and mechanical vibrations from the electromechanical device. The data acquisition and storage platform was developed in LabVIEW using National Instruments (NI) acquisition hardware.

**Figure 8:**
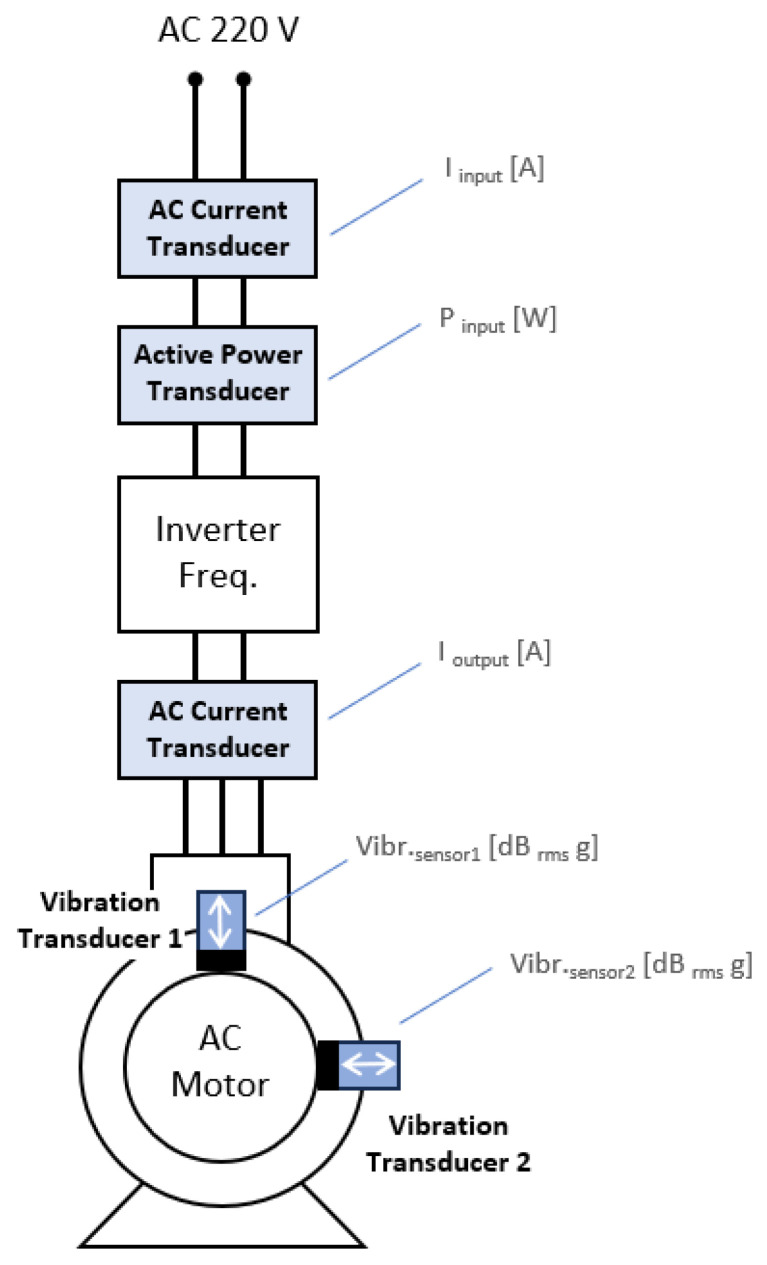
Instrumentation system: transducers’ positions and their signals.

As shown in Figure 8, a current transducer and a power transducer were utilized before the frequency inverter to capture the “I input [A]” and “P input [W]” signals, respectively. Another current transducer was employed after this motor drive to acquire the “I output [A]” signal. Additionally, the accelerometers (mechanical vibration transducers) were arranged at 90° angles to each other, with one of them positioned vertically at the top of the electric motor aligned with the shaft support bearing.

**Figure 9:**
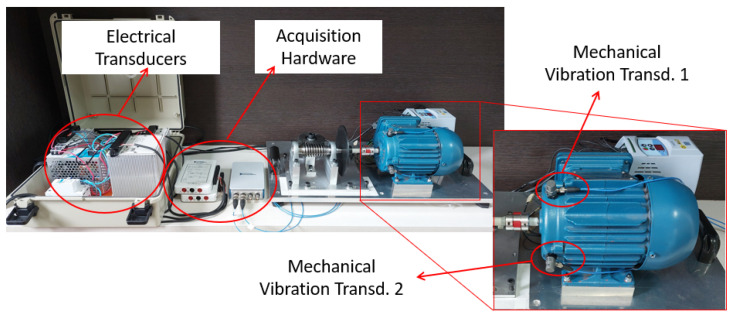
Instrumentation system: transducers and data acquisition hardware.

Figure 9 shows the details of the setup assembly of the experimental bench.

The attribute extraction and selection processes, model training, and performance evaluation and validation were implemented in Anaconda’s data science platform using the Jupyter Notebook web-based computing environment and Python language. The following libraries were used: Pandas, Numpy, Matplotlib, Sklearn, Tensorflow, and Seaborn.

### 3.2. Requirements and Data Collection

This section presents the scenarios for using the experimental bench: normal operating conditions and fault conditions. The fault conditions are detailed, as well as the signals collected for generating the corresponding datasets. With the electromechanical device, it is possible to simulate five different situations in applications driven by electric motors, here called scenarios: normal operating conditions and four anomaly scenarios.

**Figure 10:**
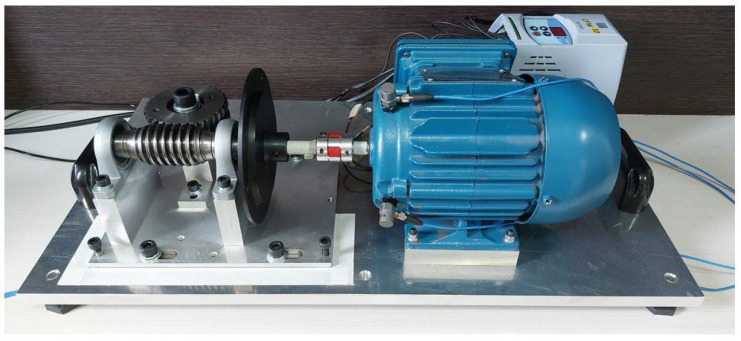
Normal.

**Figure 11:**
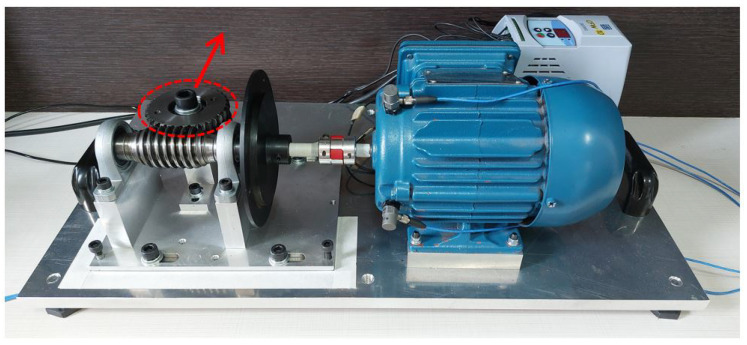
Uncoupled.

**Figure 12:**
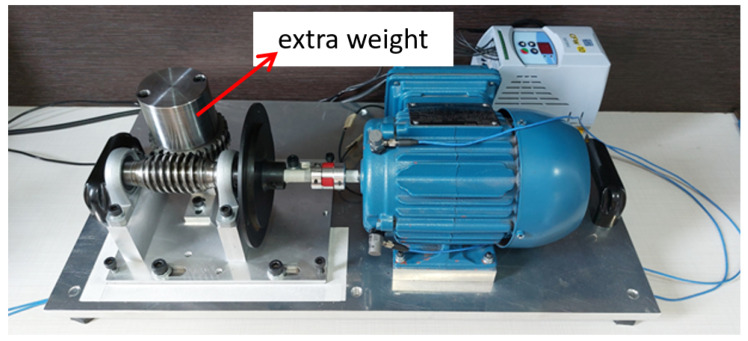
Overload.

**Figure 13:**
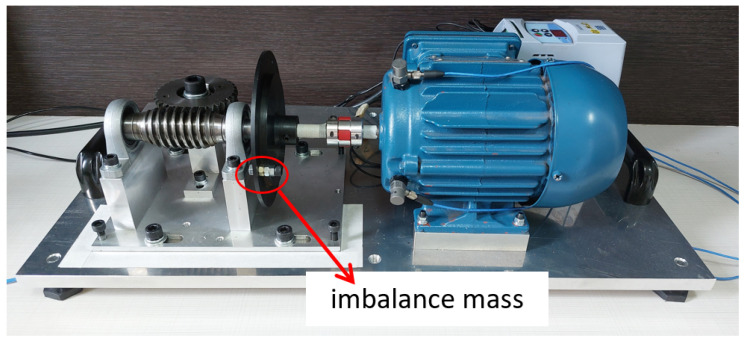
Unbalanced.

**Figure 14:**
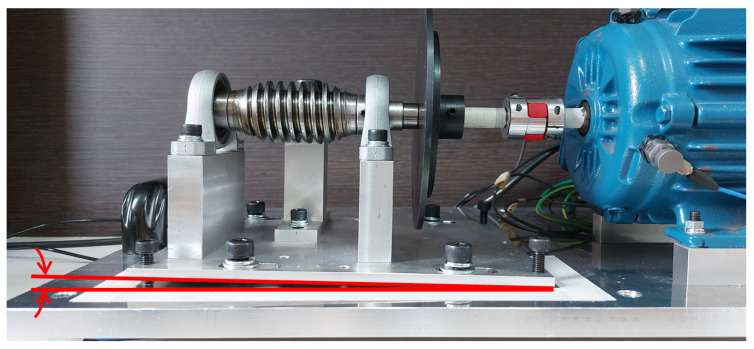
Misaligned.

The following scenarios were considered:

- Normal: Endless shaft coupling with the crown gear, without overload or imbalance and misalignment of the set driven by the electric motor (Figure 10).
- Uncoupled: Decoupling the crown gear from the endless shaft, moving the crown gear away in the direction of the red arrow, as illustrated in Figure 11. Thus, the driven shaft rotates “no load”, with no torque generated by the coupling with the crown gear. This condition simulates a possible interruption of transmission from the rotary movement of the motor shaft to the driven mechanism.
- Overload: Over the crown of the worm shaft system, an extra weight is added (±981 g), causing an increase in the torque exerted on the axis of the motor (Figure 12). This situation simulates a possible torque increase caused by, for example, problems in the mechanical transmission system.
- Unbalanced: A mass of ±8.2 g imbalance is introduced (Figure 13). This situation simulates possible imbalances in the mechanics driven by the motor shaft.
- Misaligned: Angular misalignment of the endless shaft relative to the motor shaft, as illustrated in Figure 14. This situation simulates a misalignment of the mechanical assembly of the transmission with the motor shaft.

A deliberate choice was made to abstain from introducing variations in fault intensity. While recognizing the possibility that this decision could potentially impact the overall performance of the trained models, it played a crucial role in more effectively addressing the fundamental goals of this study. By simplifying the process of fault generation, a broader spectrum of potential fault scenarios was encompassed. Consequently, this approach facilitated a more comprehensive assessment of the effectiveness of data science and machine learning algorithms in the context of anomaly detection within industrial equipment and their ability to help define the instrumentation configuration in a PdM application.

## 4. Experiments and Results

**Figure 15:**
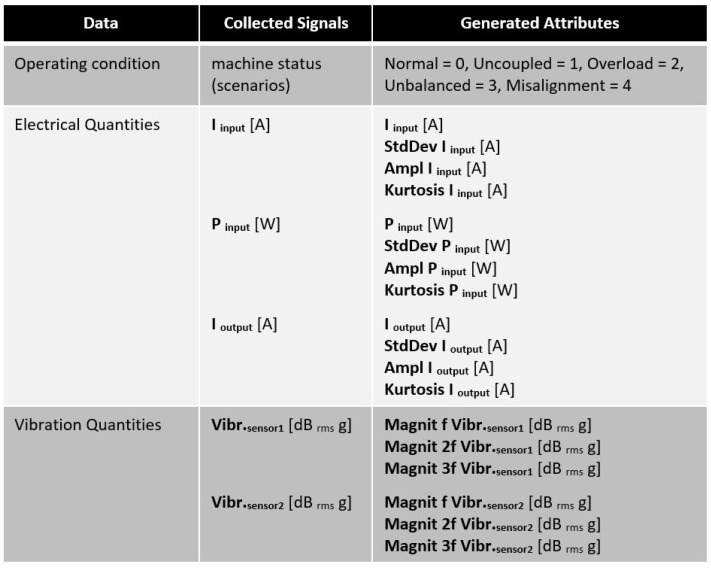
Collected signals and generated attributes.

**Figure 16:**
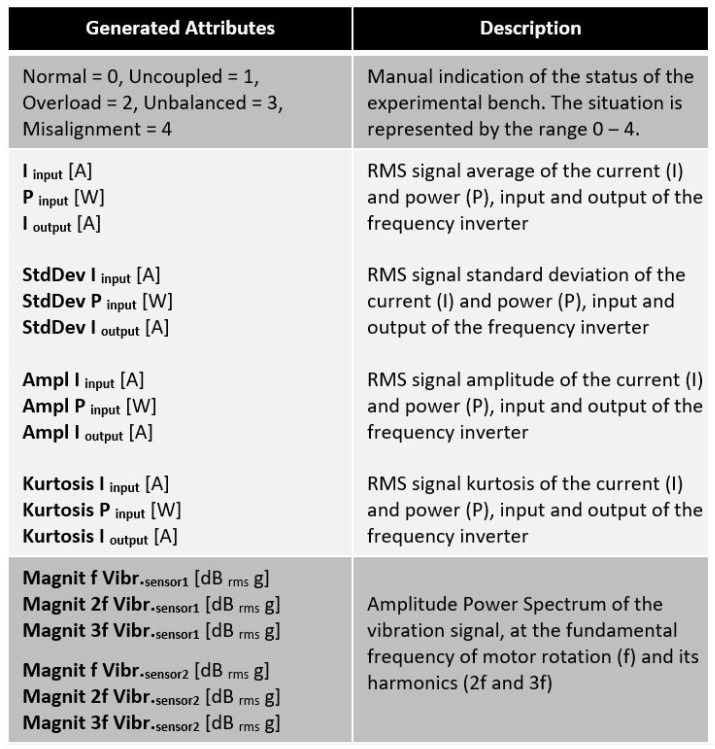
Descriptions of generated attributes.

The collected signals and generated attributes for the samples of each scenario comprised the dataset built by us that was utilized to simulate several anomaly types. They are described in Figure 15. Figure 16 presents a brief explanation of each generated attribute.

### 4.1. Data Preparation

This subsection presents the attribute extraction and reduction step to select the information most relevant to the identification of the patterns of interest.

**Figure 17:**
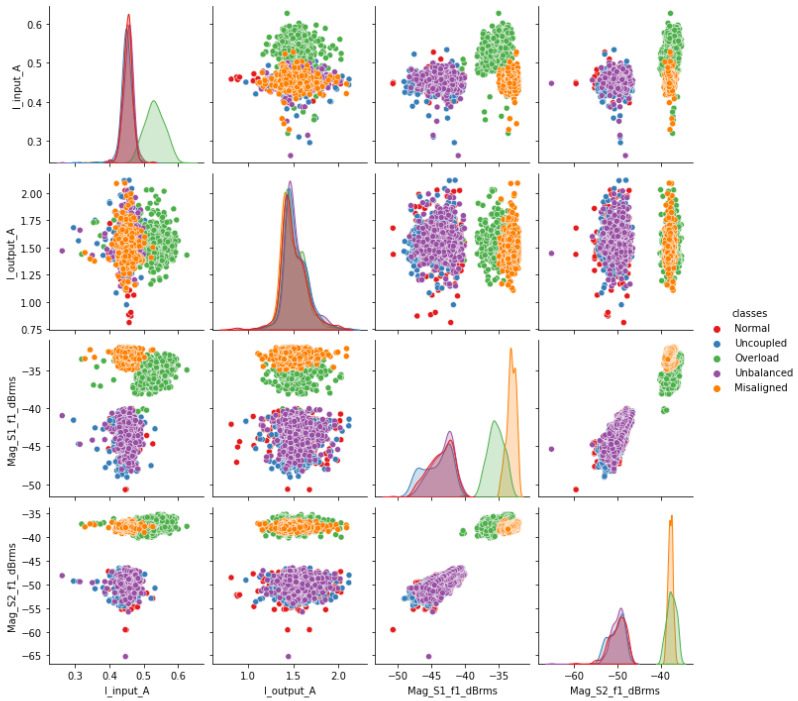
Relation between attributes and classes.

For the initial exploration, visual verification of the simple relationships between the attributes and classes (scenarios) was performed. For this task, a scatter plot matrix was generated, as shown in Figure 17. The relevant attributes were initially chosen and generated by four transducers: input current, output current, vibration sensor 1 and vibration sensor 2 at the fundamental frequency f (motor rotation). In Figure 17, we can see a certain distinction in the grouping of samples for the “misalignment” and “overload” conditions. On the other hand, there is some overlap for the “normal”, uncoupled”, and “unbalanced” patterns.

It can be concluded that apparently, the attributes appear promising for the process of identifying the patterns of the five scenarios. To this end, a selection process for attributes becomes necessary and its description is presented in the following paragraphs.

#### Attribute Selection

For the selection of attributes, some tools were adopted, such as the correlation matrix. For some attribute correlation certifications and the detection of redundancies, the following plots were generated: scatter plots and box plots.

**Figure 18:**
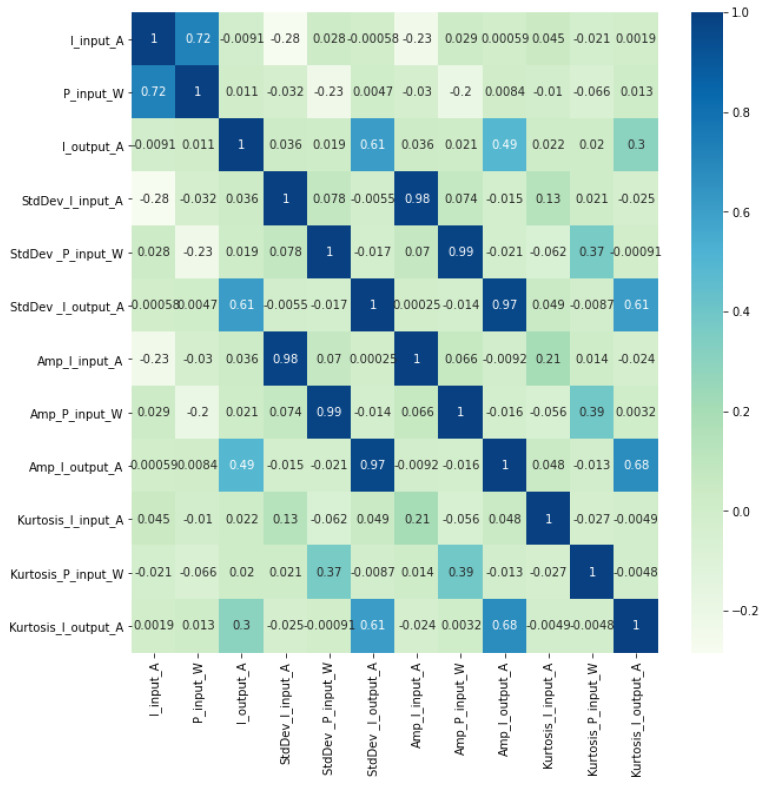
Correlation of electrical attributes.

The correlation matrix eliminated the influence of the scaling of values, thus illustrating more clearly the strength of the relationships between the attributes. Figure 18 shows the correlation matrix of the electrical attributes.

When analyzing the correlation matrix, a strong correlation between the attributes can be seen, indicating the possible redundancy of the following attributes generated from the electrical quantities:

- P_input_W;
- StdDev_I_output_A;
- Amp_I_input_A;
- Amp_P_input_W;
- Amp_I_output_A;
- Kurtosis_I_output_A.

**Figure 19:**
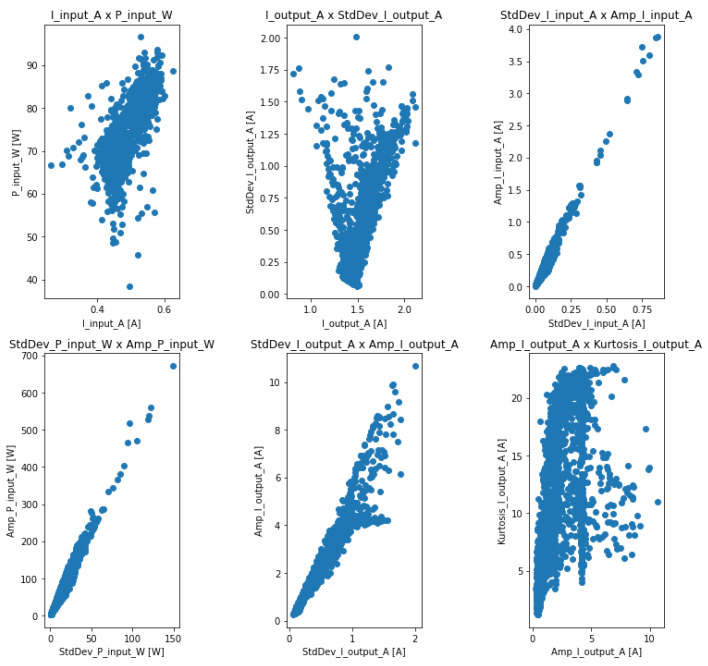
Correlations between electrical quantities.

To confirm this redundancy, scatter plots were generated for the visual verification of this correlation intensity, as shown in Figure 19.

It can be concluded that there was indeed a strong correlation between these attributes, confirming their redundancy. Thus, these attributes were excluded. We then reduced the number of attributes of the electrical quantities from 12 to 6.

**Figure 20:**
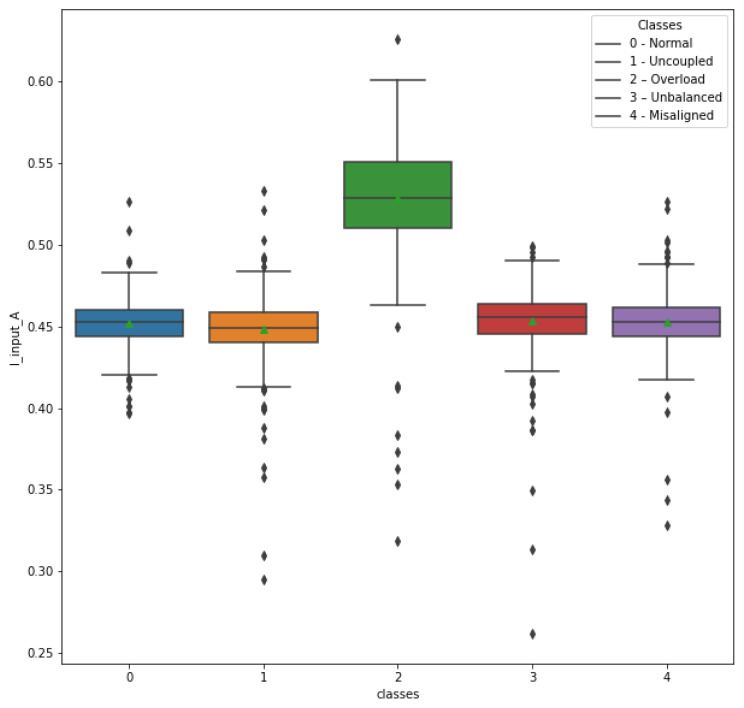
Attribute I_input_A in relation to classes.

Box plots were generated for a better understanding of the relevance of the attributes of the remaining electrical quantities. In Figure 20, it is possible to observe the behavior of the attribute I_input_A in relation to the operating conditions of the machine, that is, in relation to the classes (scenarios). Note the clear relevance of this attribute in identifying class 2 (overload), with a significant difference in the mean, median, and interval values between the quartiles in relation to the other classes. In this way, the attribute I_input_A was considered relevant.

**Figure 21:**
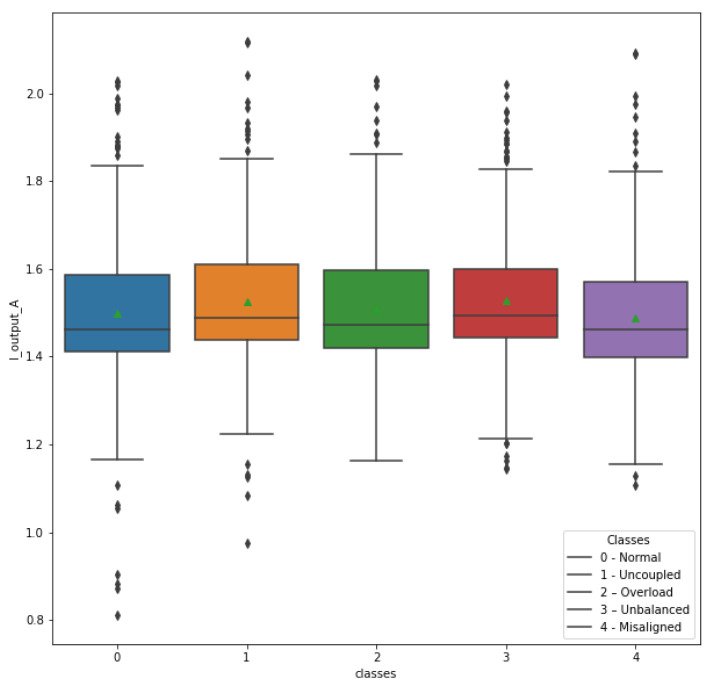
Attribute I_output_A in relation to classes.

Following the same process, a box plot illustrating the behavior of the I_output_A attribute was generated, as shown in Figure 21. Contrary to the conclusion regarding the I_input_A attribute, the I_output_A attribute does not clearly demonstrate its relevance for class identification. Therefore, it was decided to exclude this attribute and the Kurtosis_I_output_A attribute derived from the same data source: the drive output current sensor. Therefore, the consideration of the inverter output current sensor was discontinued, thereby enhancing the feasibility and cost-effectiveness of the anomaly detection system.

**Figure 22:**
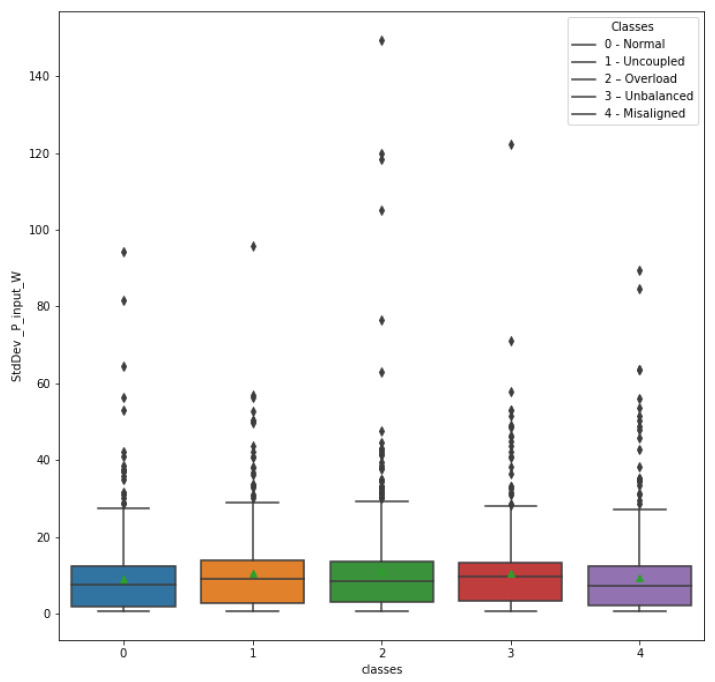
Attribute StdDev_P_input_W in relation to classes.

**Figure 23:**
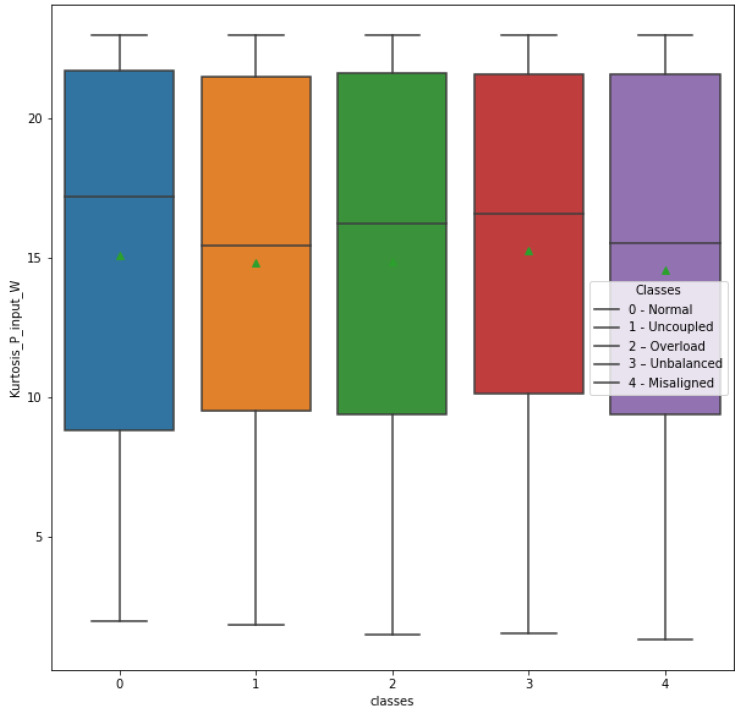
Attribute Kurtosis_P_input_W in relation to classes.

The same process was adopted for the StdDev_P_input_W and Kurtosis_P_input_W attributes, with their respective box plots shown in Figure 22 and Figure 23.

The box plot of the StdDev_P_input_W attribute exhibits many outliers. In addition, apparently, there is not a great emphasis on the variation of measures among the different classes. In this way, it was decided to exclude it from the list of attributes used in model training.

The box plot of the Kurtosis_P_input_W attribute shows a certain variation of measures in relation to the different classes, mainly in the average measure. As this attribute would require an additional input power sensor in the frequency inverter, it was decided to exclude it. This reduces the number of electrical magnitude sensors needed, contributing again to the viability and cost-effectiveness of the anomaly detection system.

Thus, the selection of attributes pertaining to electrical quantities is hereby concluded. Based on this analysis, we decided to adopt only one input current sensor in the frequency inverter, with only three attributes generated from it: I-input_A, StdDev_I_input_A, and Kurtosis_I_input_A.

Continuing the process of analyzing and excluding redundant attributes, the attributes generated by the mechanical vibration transducers were analyzed.

**Figure 24:**
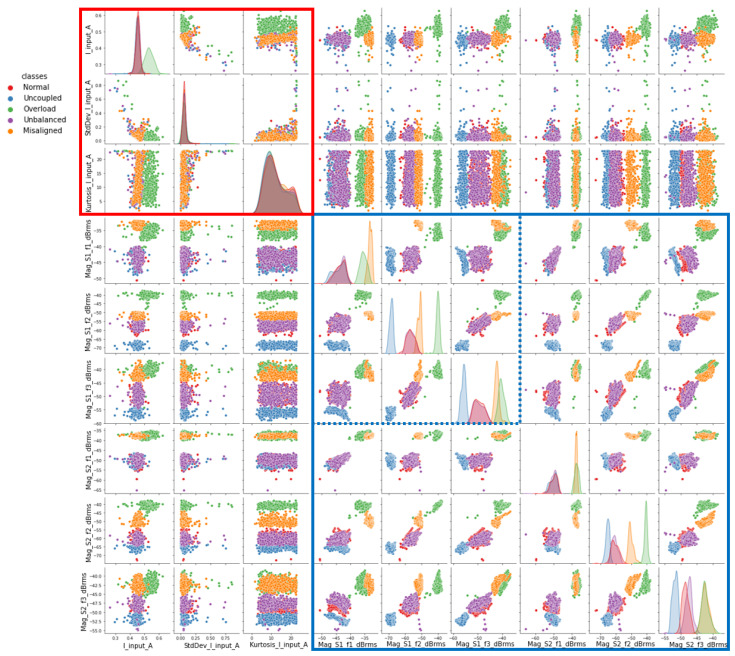
Clustering of the classes with electrical and vibration attributes.

It was decided to evaluate the behavior of the classes using the previously selected electrical magnitude attributes first, then append the vibration attributes. To this end, a pair plot matrix was generated, as shown in Figure 24.

In Figure 24, when considering only the electrical attributes (upper corner, highlighted in red), it is possible to distinguish only class 2 (overload). On the other hand, when considering only the vibration attributes (bottom corner, highlighted in blue), in addition to being able to identify class 2 (overload), an improvement in the distinction from the other classes can be observed.

**Figure 25:**
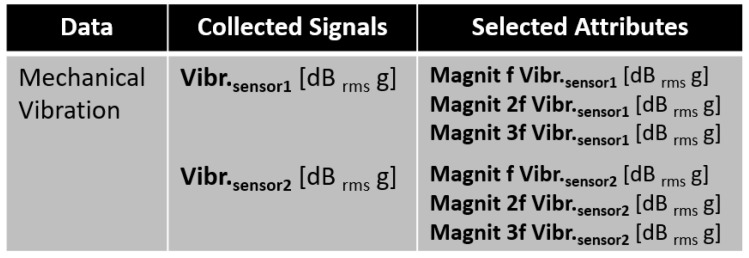
Selected attributes.

Then, it was concluded that by considering only the vibration attributes, it would be possible to distinguish all classes. After these correlation evaluations, the attributes shown in Figure 25 were retained for use in the models. Thus, from the 18 attributes generated by the five sensors, 6 attributes generated by two sensors were selected.

### 4.2. Training of the Models

In this section, the models used and the entire training process of the algorithms for the predictive tasks of detecting and diagnosing anomalies in applications driven by electric motors are presented. Guided by the literature, we chose to employ widely recognized statistical classifiers and commonly utilized learning methods to diagnose faults in rotating machinery, including k-nearest neighbor (k-NN), the naive Bayes classifier, support vector machine (SVM), and multi-layer perceptron (MLP).

The training was supervised, with balanced samples of five classes (target attributes) representing the possible situations in industrial applications driven by electric motors for rotating machines. The possible situations are identified by the “classes” attribute, with numerical values ranging from 0 to 4 (see Table 1).

**Figure 26:**
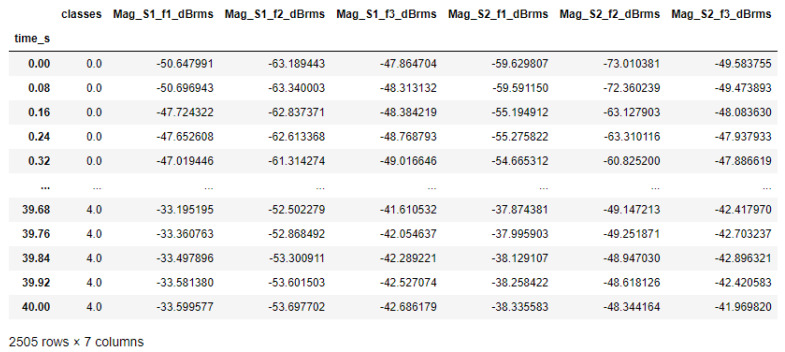
Dataset overview for training.

Figure 26 shows the dataset containing the target attributes (classes) and the predictive attributes selected in the previous section.

**Figure 27:**
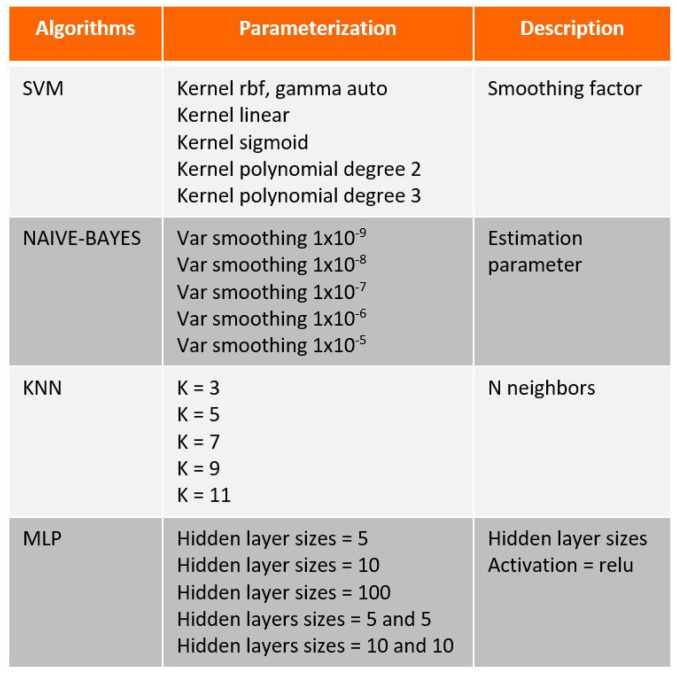
Algorithms’ parametrizations.

About 501 samples were generated for each of the five classes, totaling 2505 samples. For all trained algorithms, a cross-validation technique was adopted, dividing the samples into 75% for training (1878 samples) and 25% for testing (627 samples). Figure 27 presents the trained algorithms along with their respective parameterizations.

### 4.3. Performance Assessment and Validation

**Figure 28:**
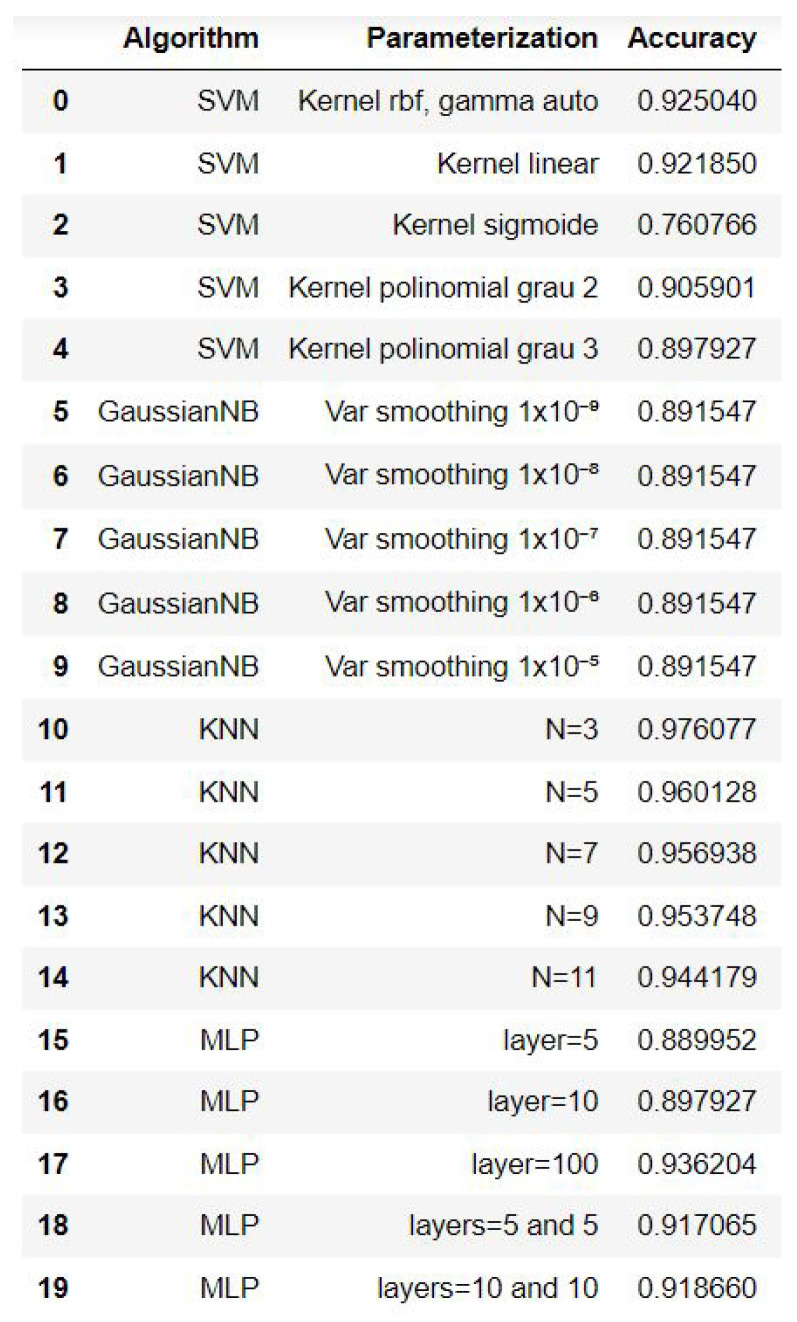
Accuracy of ML methods.

For the evaluation of the models in the different anomaly scenarios, the following metrics were used: accuracy, precision, recall, and F1 score. As a visual tool, confusion matrices were generated. Initially, all models were evaluated for their accuracy. Figure 28 displays the results.

**Figure 29:**
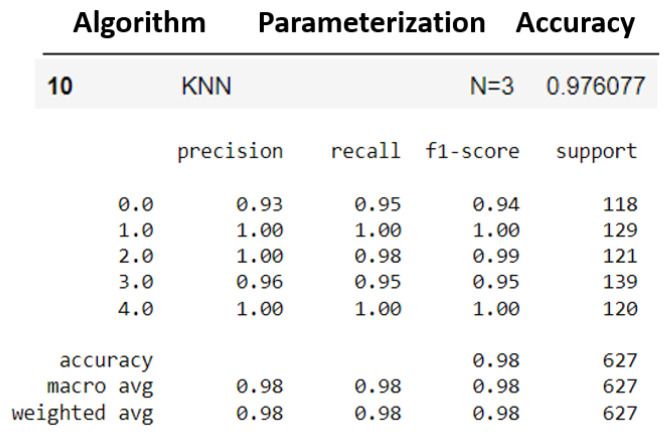
Metrics for k-NN evaluation.

**Figure 30:**
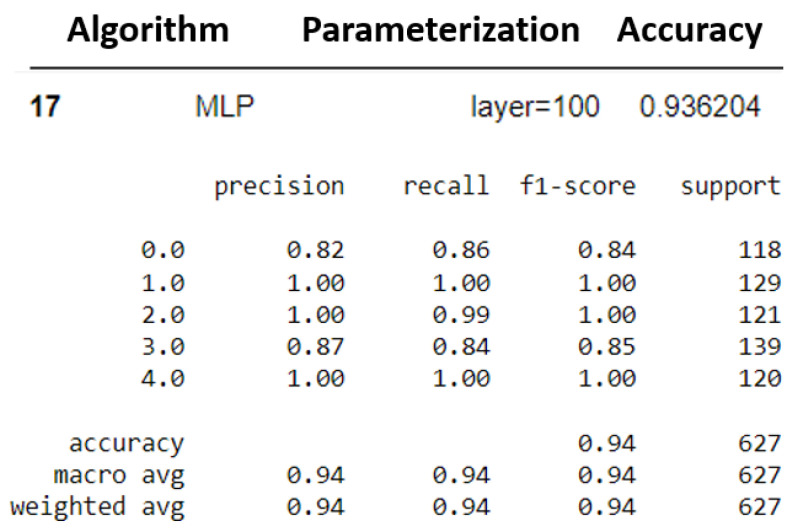
Metrics for MLP evaluation.

The two models that presented the best results were selected: k-NN (K = 3) and MLP (layers = 100). Subsequently, the other evaluation metrics were generated. Figure 29 and Figure 30 show the performance of the models for the precision, recall, F1 score, and accuracy metrics.

**Figure 31:**
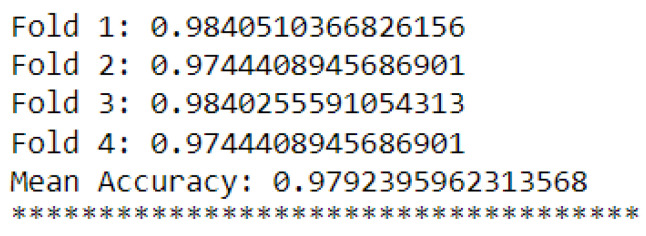
K-fold validation for k-NN.

**Figure 32:**
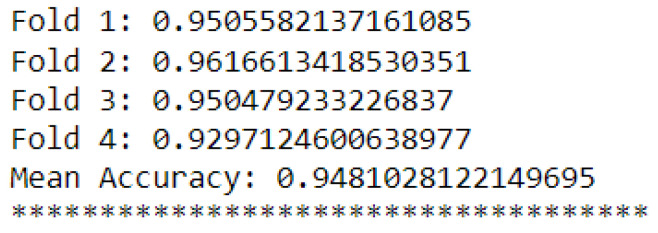
K-fold validation for MLP.

Additionally, Figure 31 and Figure 32 show their cross-validations by dividing the data into four parts (four folds).

**Figure 33:**
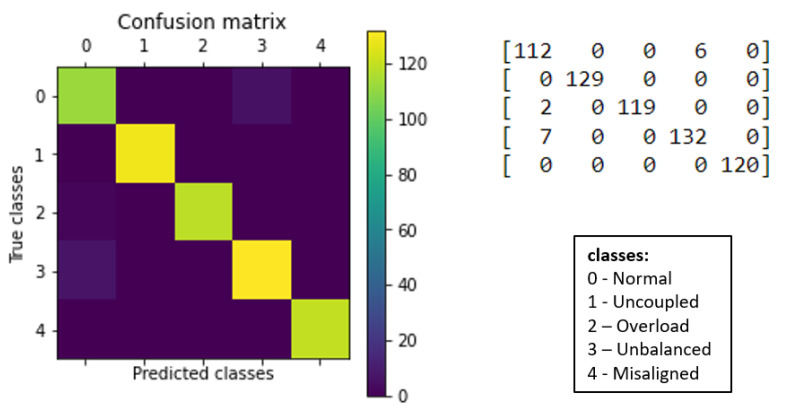
Confusion matrix for k-NN.

**Figure 34:**
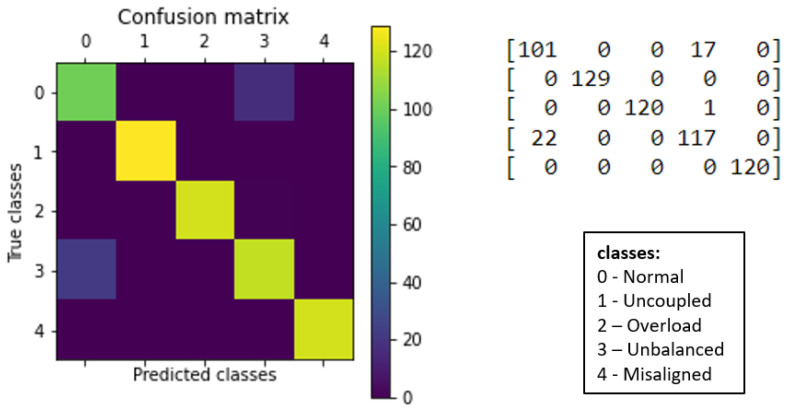
Confusion matrix for MLP.

For better visualization of the results, confusion matrices were generated for each algorithm, as presented below (see Figure 33 and Figure 34).

The k-NN model (K = 3) had a higher accuracy average than the MLP model (layers = 100) (see Figure 29 and Figure 30). Furthermore, when observing the confusion matrix for the k-NN model (K = 3) (see Figure 33), it can be seen that in a test set composed of 627 samples, the model was able to detect anomalies in the vast majority of cases. In the k-NN model (K = 3), there were only nine errors in anomaly detection: two errors in detecting the ‘overload’ anomaly and seven errors in detecting the ‘unbalanced’ anomaly. In contrast, the MLP model (layers = 100) exhibited 22 errors in anomaly detection, all of which were related to ‘unbalanced’ anomalies. The classifications of interest are those referring to the detection of anomalies, that is, the classifications of classes 1 to 4 since class 0 represents a normal situation of operation. Thus, we can consider that the best algorithm was the k-NN model (K = 3).

## 5. Conclusions and Future Work

In this article, the performance of certain machine learning methods in the context of predictive maintenance for industrial equipment was examined. Specifically, we addressed the detection of anomalies in an application powered by an electric motor and subsequently diagnosed their root causes in various scenarios. These causes included the disconnection of the drive mechanism from the load, the presence of overload, imbalance, and misalignment with the electric motor shaft. Considering the outcomes achieved through multiple tests, the identification of anomalies within electric motor-driven applications and their subsequent diagnosis, including the determination of their underlying causes among the mentioned scenarios, has become feasible. Moreover, the incorporation of data science and ML algorithms played a pivotal role in defining the instrumentation setup, thereby reducing its cost. These methodologies significantly contributed to the selection of appropriate sensors and the refinement of attribute choices. The possible limitations of this study include the fact that all achieved results were obtained through the utilization of a test bench. This test bench aimed to simulate real-world scenarios of anomalies in applications driven by electric motors to the fullest extent possible. The scope of these anomaly simulations was centered around the mechanical aspects of transmission, focusing on a worm and gear system. As a part of future work, the proposed approach can be extended to encompass other types of transmission mechanisms that are also driven by electric motors. Additionally, anomalies directly linked to the characteristics of engine electromechanics could also be explored.

## Figures and Tables

**Table 1:** Attribute classes and scenarios.

Class	Scenario
0.0	Normal
1.0	Uncoupled
2.0	Overload
3.0	Unbalanced
4.0	Misaligned

## Data Availability

Data are contained within the article.
